# Early-life adversity and later-life mental health: a conditional process analysis of sense of coherence and resilience-related resources

**DOI:** 10.3389/frcha.2023.1213142

**Published:** 2023-08-16

**Authors:** Shauna L. Rohner, Florence Bernays, Andreas Maercker, Myriam V. Thoma

**Affiliations:** ^1^ Competence Centre for Mental Health, Department of Health, OST—University of Applied Sciences of Eastern Switzerland, St. Gallen, Switzerland; ^2^ University Research Priority Program “Dynamics of Healthy Aging”, University of Zurich, Zurich, Switzerland; ^3^ Department of Business Administration, Chair of Human Resource Management and Leadership, University of Zurich, Zurich, Switzerland; ^4^ Psychopathology and Clinical Intervention, Institute of Psychology, University of Zurich, Zurich, Switzerland

**Keywords:** early-life adversity, sense of coherence—revised, resilience-related resources, mental health and wellbeing, moderated mediation

## Abstract

**Objective:**

While early-life adversity can have negative effects on health and wellbeing that persist across the lifespan, some individuals show indications of resilience. Resilience can be understood as a dynamic coping process involving the mobilization of resources in response to adversity exposure. Sense of coherence—revised (SOC-R), an ability linked to health maintenance in the face of adversity, may be influential in this process. However, research is lacking on the mechanisms underpinning SOC-R and resilience-related resources and their impact on the (mental) health of individuals exposed to early-life adversity. Therefore, this study examined the role of SOC-R and selected resilience-related resources in the relationship between early-life adversity and later-life health and wellbeing.

**Method:**

Participants were *N* = 531 Irish (older) adults (58.2% female, mean age = 59.5 years, range = 50–86 years). Standardized questionnaires assessed retrospective reports of early-life adversity, as well as current physical and mental health, satisfaction with life, SOC-R, and resilience-related resources (self-efficacy, optimism, social support). A multiple mediation analysis tested the indirect effects of the resources and a moderated mediation tested for conditional dependence on SOC-R.

**Results:**

For mental health and satisfaction with life, significant partial mediations were found for all three resources. Only optimism showed a significant partial mediation for physical health. In the moderated mediation, SOC-R significantly moderated the associations between early-life adversity and self-efficacy (*b *= .06, *t* = 3.65, *p* = .001), optimism (*b *= .04, *t* = 2.60, *p* = .009), and social support (*b* = .08, *t* = 3.75, *p* < .001). The indirect effects were larger at high rather than low SOC-R, indicating that the mediating effects of the resources were greater for individuals with a stronger SOC-R.

**Conclusion:**

A strong SOC-R may have a beneficial influence on health and wellbeing by mitigating the detrimental effect of early-life adversity on the resources self-efficacy, optimism, and social support. Future avenues for research include the expanded assessment of resources and the potential role of SOC-R in successful ageing through the selection and adaptation of goals and resources into older age. SOC-R may represent a promising target for psychotherapeutic interventions promoting resilience in survivors of early-life adversity.

## 1. Introduction

Trauma or adversity in childhood and adolescence can take many forms, including abuse, neglect, extreme poverty, parental loss, and domestic or community violence (1). As childhood and adolescence represent a stress-sensitive period of physiological development, such early-life adversity can have long-lasting effects on the physiological systems that regulate the stress-response, with negative implications for health and wellbeing that can persist across the lifespan [e.g., (2, 3)]. For instance, a recent systematic review and meta-analysis of 23 longitudinal cohort studies examined the link between exposure to early-life adversity and adult-diagnosed depression, anxiety, psychotic disorder, or bipolar disorder (4). Significant associations were found between various indicators of early-life adversity and the diagnosis of a mental health disorder in later adulthood, including physical neglect (OR = 1.93, 95% CI 1.31–2.85), being a victim of bullying (OR = 2.36, 95% CI 1.45–3.86), and multiple trauma exposure (OR = 3.11, 95% CI 1.36–7.14). Similarly for physical health, studies of individuals with and without childhood trauma exposure indicate that those who experienced early-life adversity report worse physical health outcomes in middle adulthood [e.g., higher cardiometabolic risk (5); more self-reported health problems and lower perceived quality of physical health (6)]; as well as in older adulthood [e.g., higher incidence of diabetes, cardiovascular problems, and stroke (7)]. Such research not only highlights the link between early-life adversity and later life (psycho)pathology, but also hints at the broader consequences, such as the associated burden and costs on health and social care systems (8).

Despite this, not all those who experience early-life adversity display clinically relevant symptomatology. For instance, a recent study with Swiss older adult survivors of child welfare-related maltreatment found that despite severe and often prolonged early-life adversity, approximately 30% of survivors did not meet the full diagnostic criteria for a current or lifetime DSM-5 disorder (9). In addition, a longitudinal prospective study in the United States assessed children who were at risk or exposed to child maltreatment and found that 48% still demonstrated adaptive functioning across behavioral, social, and developmental domains (10). Similarly, a recent study by Yoon et al. (11) assessed a high-risk sample of children involved in the child welfare system in the United States. Findings showed that 56% could be classified into a multi-domain resilience profile, exhibiting positive adaptation and competence across all domains of cognitive, emotional, social, and behavioral functioning. Evidence of these heterogenous (mental) health outcomes following early-life adversity points to the potential for resilience, referring to relatively stable, healthy levels of psychological and physical functioning despite significant stress, trauma, or adversity (12). For instance, seminal research on resilience identified positive adjustment or adaptive trajectories in children exposed to significant adversities, including war, terrorism, disasters, and maltreatment (13–15).

Theoretical models of resilience, such as the multi-system model of resilience [MSMR (16)], or the socio-interpersonal model of trauma sequalae (17), view resilience as a dynamic coping process involving the mobilization and interaction of resources in response to adversity exposure. The MSMR suggests that resilience capacity is shaped by multiple resources, including traits, psychological personality-correlates, protective factors, and social and community structures (16). Applying this to trauma and adversity, the socio-interpersonal model depicts these resources on the level of the individual, close relationships, and distant social contexts. It proposes that interactions between and across these levels determine how adversity is mitigated or intensified, which in turn, influences response outcomes (17). The application of these conceptual frameworks to empirical data can aid in the identification of key resilience-related resources in the relationship between early-life adversity and later-life health and wellbeing.

These protective or promotive resources can be broadly categorized into positive resources within the individual, such as self-efficacy, and positive resources external to the individual, such as social support (16, 18). With regard to early-life adversity, research on individual attributes or internal resilience resources has identified several key characteristics or personality traits (e.g., self-efficacy, mastery, hardiness, persistence, emotional reactivity) that may help buffer against negative outcomes (19). For example, a study of protective factors in children exposed to maltreatment in the home identified optimism, self-efficacy, and adaptability as key individual resources linked to fewer posttraumatic stress disorder (PTSD) symptoms (20). Regarding later-life mental health, a longitudinal population-based birth cohort analysis in the United Kingdom by Cosco and colleagues (21) examined resilience resources in older adults with a history of early-life adversity. Results identified individual and social resources (e.g., education, social support, neighborhood cohesion) associated with lower levels of mental distress in later life. These resources partly mediated the relationship between early-life adversity and later-life mental distress, with the effect of social support being the greatest (21).

Furthermore, systematic reviews on resilience and protective factors in individuals with a history of early-life adversity provide support for several individual and external resources linked to better health and wellbeing [e.g., self-efficacy; optimism; coping skills; interpersonal competence or the ability to interact or work with others; and social support in the form of practical, tangible, or emotional assistance or comfort from family, peers, community, or significant others (22, 23)]. However, these individual and external resources do not act in isolation, but rather interact to impact (mental) health outcomes (16, 24). Although emerging research has attempted to characterize the links between early-life adversity and the expression of psychopathology or resilience [e.g., (25, 26)], the interplay of relevant mechanisms remains largely unclear. Ongoing research attention is therefore required to clarify the mechanisms underpinning the pathways to (mental) health and wellbeing following early-life adversity. A better understanding of the mechanistic pathways could help identify whether, and how, the negative (mental) health consequences of early-life adversity could be mitigated or prevented, and adaptive outcomes promoted.

One resilience-related aspect that may be influential in this process is sense of coherence (SOC). The original SOC concept developed by Antonovsky (27) suggests that the way in which individuals view their lives can have an influence on their health. This concept was further refined by Bachem and Maercker (28), with a sense of coherence—revised (SOC-R) describing an individual's ability to perceive, integrate, and balance positive and negative life experiences in order to maintain their health and wellbeing. The SOC-R construct is comprised of three theoretical dimensions, with a focus on health maintenance and dealing with the ambiguity of challenges: Managing and dealing with difficult situations (manageability), balancing positive and negative experiences and feelings (balance), and considering different perspectives and understanding connections (reflection) (28). SOC-R may play a key role in maintaining health and wellbeing after exposure to early-life adversity, as it is proposed to develop and strengthen early in life by successfully overcoming challenging experiences. Consequently, according to the theoretical assumptions of SOC-R, individuals with a strong SOC-R are assumed to be better at utilizing available and appropriate resources to overcome stress and adversity in later life (28, 29).

Previous research, mainly with the original SOC concept, has demonstrated associations with key resilience-related resources and better (mental) health outcomes. For instance, research examining predictors of distress in patients with cancer found that a stronger SOC was associated with higher levels of optimism, which was linked to fewer symptoms of depression and anxiety than those with weaker SOC and lower levels of optimism (30). In addition, research by Wiesmann and colleagues (31) on the experience of bodily pain found that the resources self-esteem, self-efficacy, optimism, and social support had an indirect effect on pain through SOC. A recent study by Sölva and colleagues (32) used latent class analysis to identify distinct classes of adaptation in trauma-exposed children and adolescents in residential care in Austria. Results revealed a resilient class characterized by lower levels of symptom severity (i.e., dissociative symptoms, internalizing and externalizing problems, interpersonal problems, and thought problems); as well as highest levels of the protective factors SOC, self-efficacy, peer support, and caregiver social and emotional support (32). While such research indicates the potential protective influence of SOC, few studies have examined the interaction of resilience-related resources, particularly not with the more recently refined SOC-R.

Existing research on SOC-R has mainly established its role in the relationship between adversity and health outcomes. For example, research by Thoma and colleagues (33) with a representative German sample identified a moderating effect of SOC-R in the relationship between trauma exposure and mental health. A strong SOC-R was associated with lower depression scores compared to those with a weaker SOC-R, even at high levels of childhood neglect and lifetime traumatic events (33). In addition, a study on emergency medical service rescue workers who were regularly exposed to potentially traumatic on-duty events found that a higher SOC-R was associated with less post-traumatic, depressive, and somatic symptoms (34).

Initial studies focusing on SOC-R in adult and older adult samples have demonstrated a moderating or mediating influence in the relationship between adversity, stress, and mental (health) outcomes. For instance, a study with Swiss older adults identified the SOC-R manageability subscale as a significant moderator, with higher SOC-R manageability linked to better general mental health across all levels of chronic stress (35). Similarly, recent research with Irish older adults found that those with a stronger SOC-R reported better mental health, even at high levels of acute perceived stress (36). As a next step, research is needed that incorporates key resilience-related resources into the model with SOC-R to obtain a more complete picture of the mechanisms underpinning health and wellbeing following exposure to early-life adversity.

It was therefore the aim of this study to examine the role of SOC-R and resilience-related resources in the relationship between early-life adversity and later-life health and wellbeing. To select key resilience-related resources that encompass a range of individual protective factors, psychological personality-correlates, and socio-interpersonal factors, conceptual frameworks and theoretical models of resilience were first consulted [i.e., (16, 17)]. These factors were further refined for relevance to the study sample and research questions by drawing on empirical studies in individuals with a history of early-life adversity [e.g., (21–23, 32)], to identify resources commonly linked to adaptive outcomes or resilience. As a result of this process, self-efficacy, optimism, and social support were selected as key internal and external resources for inclusion into the model with SOC-R. Given that individuals with a strong SOC-R should be better able to utilize their resources to successfully overcome adversity (28, 29); a moderated mediation model was assessed with SOC-R as the moderator. This tested whether the mediation effects of the resources differed depending on the level of SOC-R. Specifically, it was hypothesized that the mediating effects of the resources would be greater for participants with a stronger SOC-R.

## 2. Materials and methods

### 2.1. Study design and procedure

This study assesses data from a cross-sectional quantitative questionnaire survey, conducted in Ireland between June and December 2018 as part of the larger Swiss National Science Foundation (SNSF) funded project “Differential aging trajectories in high-risk individuals with past experiences of early adversity”, within the SNSF National Research Program 76 “Welfare and Coercion—Past, Present, and Future” (http://www.nrp76.ch/en). The study was conducted by a research team of the Psychological Institute at the University of Zurich, in collaboration with University College Dublin, National College of Ireland, and Ulster University. The study procedure was approved by the Ethics Committee of the Faculty of Arts and Social Sciences in the University of Zurich, Switzerland (ID 18.6.1), and the Human Research Ethics Committee—Humanities in University College Dublin, Ireland (ID HS-18-30-Carr). Written informed consent was provided by all participants in accordance with the Declaration of Helsinki.

### 2.2. Participants and procedure

Eligible participants were Irish individuals, native English speakers, and aged 50 years or older. The sample size of 531 was considered sufficient to conduct the moderated mediation analyses based on the established guidelines of a minimum of 5–10 observations per estimated parameter (37), the minimum of 200 participants for mediation analyses (38), and the sample sizes of previous empirical studies using moderated mediation analyses [e.g., (39)]. Recruitment methods included flyers posted in public and community spaces (e.g., libraries, adult education centers), online advertisements, radio interviews with the study lead (first author), and networks of the study collaborators. Interested individuals contacted the study team by email or telephone and were screened for eligibility. Qualtrics survey management software was additionally used to recruit the target population and reduce sampling or self-selection biases (40). This helped to ensure a more equal distribution of cohort characteristics, such as age, gender, socio-economic status, and education level. For representativeness, the sample from each research panel was proportioned to the general population and then randomized. Some participants did not meet the inclusion or quality check criteria and were screened out, resulting in an overall response rate of 97.5%. Eligible participants could either complete the paper-pencil questionnaire survey or the online survey programmed with Unipark software (41). The questionnaire survey consisted of the information sheet, the informed consent form, the study questionnaires (randomized to avoid sequence and order effects), a debriefing sheet, and a list of psychosocial support options.

### 2.3. Measures

#### 2.3.1. Socio-demographic information

Participants first completed a questionnaire to collect socio-demographic information, including age, gender, relationship status, highest level of education, and employment status.

#### 2.3.2. Sense of coherence—revised

The SOC-R scale assessed sense of coherence—revised, i.e., the way individuals perceive, integrate, and balance positive and negative life experiences in order to maintain health and wellbeing (28). Built on three theoretical dimensions of manageability, balance, and reflection, the SOC-R scale consists of 13 items rated on a five-point Likert scale (from 1 = “not at all true” to 5 = “extremely true”). It yields a total score, with higher scores indicating higher SOC-R. Previous studies have shown convergent and discriminant validity through moderate correlations with measures of mental health, such as depression (*r* = −.46, *p* < .05), anxiety (*r* = −.33, *p* < .05), and prolonged grief (*r* = −.48, *p* < .05); as well as good internal consistencies of between α = .75 and α = .87 (28, 33, 42). A good internal consistency of α = .86 was shown in the current study.

#### 2.3.3. Early-life adversity

The Adverse Childhood Experiences—International Questionnaire [ACE-IQ, (1)] assessed the following categories of early-life adversity experienced up until 18 years old: Physical, emotional, or sexual abuse; physical or emotional neglect; violence against household members; living with household members who were substance abusers, who were mentally ill or suicidal, or who were imprisoned; having one or no parents, parental separation, or divorce; bullying; community violence; and collective violence. Each early-life adversity category was scored as 0 = “no” or 1 = “yes”, resulting in a total score ranging from 0 to 13, with higher scores indicating higher levels of exposure to early-life adversity (1). Previous research has shown good discriminant validity (*F*-value = 13.90, *p* < 0.001), good convergent validity with the Childhood Trauma Questionnaire—Short Form (*r* = 0.85, *p* < 0.001), and a good internal consistency of α = .85 (43). An acceptable internal consistency of α = .70 was shown in the current study.

#### 2.3.4. Health and wellbeing

##### 2.3.4.1. Physical and mental health

The 36-item Short Form Health Survey version 1 (SF-36) assessed physical and mental health (44). Summary scores were calculated for physical health (physical component summary; PCS) and mental health (mental component summary; MCS) using eight weighted subscales: Physical functioning, bodily pain, role limitations due to physical health problems, role limitations due to emotional problems, emotional wellbeing, social functioning, energy/fatigue, general health perceptions (44). Studies have shown good construct validity for the SF-36 in groups with long- and short-term illnesses, as well as good internal consistencies of α = .92 for PCS and α = .91 for MCS (45, 46). Acceptable internal consistencies of α = .74 (PCS) and α = .77 (MCS) were shown in the current study.

##### 2.3.4.2. Satisfaction with life

The Satisfaction with Life Scale (SWLS) was used as an index of subjective wellbeing with regard to overall quality of and satisfaction with life (47). It consists of five items, rated on a seven-point Likert scale (from 1 = “strongly disagree” to 7 = “strongly agree”), with higher scores indicating higher life satisfaction. The SWLS has previously shown convergent validity through moderately strong correlations with other measures of subjective wellbeing, such as happiness (*r* = .58) and affect balance (*r* = .50); as well as a good internal consistency of α = .87 (47). A good internal consistency of α = .91 was shown in the current study.

#### 2.3.5. Resources

##### 2.3.5.1. Self-efficacy

The General Self-Efficacy Scale (GSE) assessed perceived self-efficacy in coping with difficult life circumstances (48). It consists of 10 items, rated on a four-point Likert scale (from 1 = “not at all true” to 4 = “exactly true”). It yields a total score, with higher scores indicating higher perceived self-efficacy. The GSE has shown convergent and discriminant validity through moderate to strong correlations with measures such as anxiety (*r* = −.43) and optimism (*r* = .60); as well as an acceptable to good internal consistency of between α = .75 and α = .91 (49). A good internal consistency of α = .84 was shown in the current study.

##### 2.3.5.2. Optimism

The Life Orientation Test—Revised (LOT-R) assessed the level of dispositional optimism in relation to expectations about one's future (50). It consists of six items (plus four filler items), rated on a four-point Likert scale (from 0 = “strongly disagree” to 4 = “strongly agree”). It yields a total score, with higher scores indicating higher levels of optimism. The LOT-R has shown convergent and discriminant validity through moderate to strong correlations with measures such as self-mastery (*r* = .48), self-esteem (*r* = .50), and trait anxiety (*r* = −.53); as well as an acceptable internal consistency of α = .78 (50). A good internal consistency of α = .85 was shown in the current study.

##### 2.3.5.3. Social support

The short form of the Interpersonal Support Evaluation List (ISEL-12) assessed perceived social support (51). It consists of 12 items, rated on a four-point Likert scale (from 0 = “definitely false” to 3 = “definitely true”). The ISEL-12 yields a total score, with higher scores indicating higher perceived social support. Previous studies have shown convergent validity through moderate correlations with measures such as social network integration (*r* = .33) and life engagement (*r* = .40); as well as good internal consistency of between α = .80 and α = .90 (52, 53). A good internal consistency of α = .87 was shown in the current study.

#### 2.3.6. Data analysis

Statistical analyses were performed using R version 3.6.2. Less than 1% missing values were observed on a (sub)scale level, such that the average proportion of missingness across participants was 0.005%. A multiple iterative imputation technique, including predictive mean matching, was used to impute missing values by applying a chained random forest algorithm (5,000 trees calculated) using the package “missRanger”, which is a suitable algorithm for imputing mixed-type data, while maintaining variability on a realistic level (54). A multiple imputation technique was used as this method has been shown to produce more accurate and less biased estimates as it accounts for uncertainty in the data while also using less computational power compared to other techniques (55). It is also necessary to consider the complexity of the method and check that the amount of missingness is not substantial to ensure that the imputed data does not considerably influence the results and conclusions. Influential outliers, defined by a Cook's distance value greater than four times the mean (56), were analyzed for all outcome variables. Three observations were within this range, but as the results did not differ when excluding these observations, they were included in the final analysis.

##### 2.3.6.1. Moderated mediation analysis

In a first step, intercorrelations of study variables were analyzed by computing Pearson's correlations coefficients. Given that empirical studies have demonstrated a mediating role of the selected internal and external resources [e.g., (21, 57)], a multiple mediation analysis was performed in a second step to test within a combined model the indirect effects of self-efficacy, optimism, and social support as parallel mediators of the relationship between early-life adversity (ACE-IQ) and the health and wellbeing indicators (i.e., physical and mental health and satisfaction with life). Following Preacher and Hayes (58), a parallel mediation model was applied using a structural equation framework (package “lavaan”), while including age and gender as covariates. According to the conditions of mediation analysis outlined by Baron and Kenny (59), to support a mediation the predictor must be significantly related to the outcome and the mediator, and the mediator must be significantly related to the outcome variable. In the case of multiple mediation, it is beneficial to test multiple mediators simultaneously in one model as the indirect effects are then separated from the effects of other mediators (60). Indirect effects were estimated using the bootstrapping technique (5,000 resamples). As indirect effects can follow a skewed distribution (61), bias-corrected and accelerated percentile confidence intervals were reported for indirect effects, as these have been shown to be more accurate than the percentile technique (62). Statistical significance of an indirect effect was assumed when the confidence interval did not contain zero. Finally, the theoretical assumptions of SOC-R suggest that it develops and is strengthened early in life by overcoming adverse experiences, and that a strong SOC-R is linked to better use of available resources (28, 29). Therefore, a moderated mediation was conducted in the third step, with SOC-R added as a moderator of the relationship between ELA and the resources, testing whether SOC-R moderated the mediations from the previous step. To do so, significant interactions must be given between the predictor (early-life adversity) and the moderator (SOC-R) to test whether the indirect effects vary at different values of the moderator. In addition, the indirect effects of the mediators (i.e., self-efficacy, optimism, social support) must differ across different levels of the moderator (SOC-R) (63, 64). Thus, to test whether mediation effects differed depending on the level of SOC-R (i.e., high vs. low), indirect effects were investigated at one standard deviation (SD) above and below the mean of SOC-R. In line with previous studies examining the link between early-life adversity and (mental) health [e.g., (65)], age and gender were included as covariates in all equations to support the robustness of the findings irrespective of individual characteristics. Corrections for multiple comparisons were not applied as this may inflate the risk of Type II error and is rather advised for confirmatory studies (66). Furthermore, predictors were mean-centered for all equations to facilitate the interpretation of estimates. *P*-values < .05 were considered statistically significant. To test for potential multicollinearity, Variance Inflation Factors (VIF) were calculated for all predictor variables in the model. As all VIF were below the value of 4 (no VIF exceeded the value of 1.7), multicollinearity was not considered an issue in the present results (67).

## 3. Results

The following analyses examine the role of sense of coherence—revised (SOC-R) and resilience-related resources in the relationship between early-life adversity (ELA) and later-life health and wellbeing. A moderated mediation model tests whether the mediation effects of the resources differ depending on the level of SOC-R (i.e., the moderator). Specifically, it was hypothesized that in the relationship between early-life adversity and later-life (mental) health and wellbeing, the mediating effects of the resources would be greater for participants with a stronger SOC-R.

### 3.1. Sample characteristics

A total of 532 participants were recruited, with one participant excluded due to high missingness (>40%), which was above the recommended threshold of less than 10% missingness (68). The final sample consisted of *N* = 531 participants, 58.2% female (*n* = 310), with a mean age of 59.5 years (*SD* = 7.1, range = 50–86 years). The majority of the sample was married (*n* = 337, 63.5%), indicated university as their highest level of education (*n *= 240, 45.2%), and were employed (*n* = 262, 49.3%). See Table 1 for an overview of the sample characteristics.

**Table 1:** Sample characteristics.

Sample characteristics	(*N* = 531)					
	Total		Male		Female	
	*M*	*SD*	*M*	*SD*	*M*	*SD*
Age (years; age range = 50–86)	59.53	7.14	60.61	6.95	58.76	7.18
** **	*n*	(%)	*n*	(%)	*n*	(%)
Gender	**–**	**–**	221	41.6	310	58.4
Relationship status						
Single	72	13.6	30	13.6	42	13.6
In a relationship	18	3.4	6	2.7	12	3.9
Married	337	63.5	154	69.7	183	59.0
Separated/divorced	74	13.9	25	11.3	49	15.8
Widowed	30	5.6	6	2.7	24	7.7
Highest level of education						
No formal education	4	0.8	3	1.4	1	0.3
Primary school	18	3.4	7	3.2	11	3.5
Secondary/high school	167	31.5	87	39.4	80	25.8
Post-secondary training	54	10.2	18	8.1	36	11.6
Professional qualification	32	6.0	16	7.2	16	5.2
University degree	240	45.2	86	38.9	154	49.7
Other	16	3	4	1.8	12	3.9
Employment status						
Employed	262	49.3	104	47.1	158	51.0
Unemployed	113	21.3	40	18.1	73	23.5
Retired	142	26.7	73	33.0	69	22.3
Other	14	2.6	4	1.8	10	3.2

*M*, mean; *SD*, standard deviation.

### 3.2. Correlation analysis

Intercorrelations of the study variables are shown in Table 2. The majority of coefficients indicated moderate to strong correlations among study variables. For self-efficacy, Pearson's correlation coefficients suggested a small but significant negative association with early-life adversity (ELA) (*r* = −.13, *p* = .004), a small but significant positive association with physical health (*r* = .11, *p* = .013), and significant moderate and positive associations with mental health (*r* = .49, *p* < .001) and satisfaction with life (*r* = .44, *p* < .001). For optimism, a small but significant negative correlation was also found with ELA (*r* = −.21, *p* < .001), a small but significant positive correlation with physical health (*r* = .17, *p* < .001), and significant moderate and positive associations with mental health (*r* = .48, *p* < .001) and satisfaction with life (*r* = .52, *p* < .001). For social support, a small but significant negative correlation was also found with ELA (*r* = −.27, *p* = .004), a small but significant positive association with physical health (*r* = .11, *p* = .008), and significant moderate and positive associations with mental health (*r* = .49, *p* < .001) and satisfaction with life (*r* = .48, *p* < .001). Furthermore, significant correlations of small to moderate size were also found between ELA and the health and wellbeing indicators (physical health: *r* = −.26, *p* < .001; mental health: *r* = −.38, *p* < .001; satisfaction with life: *r* = −.27, *p* < .001). This enabled the testing of self-efficacy, optimism, and social support as mediators of the corresponding relationships between ELA and the health and wellbeing indicators. To ensure that possible deviations from normality did not bias the results of the correlation analysis, Spearman's rank correlations were also calculated for all of the above relationships, revealing that the results did not differ from the Pearson's correlation analysis.

**Table 2:** Means, standard deviations, and intercorrelations of study variables.

Study variables	*M*	*SD*	(1)	(2)	(3)	(4)	(5)	(6)	(7)	(8)
1. Early-life adversity (ACE-IQ)	5.3	1.4	–							
2. Self-efficacy (GSE)	30.0	5.9	−.13*	–						
3. Social support (ISEL-12)	23.2	7.6	−.27*	.44*	–					
4. Optimism (LOT-R)	14.4	5.0	−.21*	.59*	.52*	–				
5. Physical health (PCS)	48.3	11.0	−.26*	.11*	.11*	17*	–			
6. Mental health (MCS)	46.5	10.9	−.38*	.49*	.49*	.48*	.08	–		
7. Satisfaction with life (SWLS)	23.2	7.2	−.27*	.44*	.48*	.52*	.25*	.58*	–	
8. Sense of coherence—revised (SOC-R)	45.1	8.6	.05	.58*	32*	.41*	−.03	.26*	.25*	–

ACE-IQ, adverse childhood experiences—international questionnaire; GSE, general self-efficacy scale; ISEL-12, 12-item short form interpersonal support evaluation list; LOT-R, life orientation test—revised; *M*, mean; MCS, mental component summary; PCS, physical component summary; *SD*, standard deviation; SOC-R, sense of coherence—revised; SWLS, satisfaction with life scale.

* *p* < .05.

### 3.3. Mediation analysis

A multiple mediation analysis was first conducted to investigate whether self-efficacy, optimism, and social support mediated the relationships between ELA and the health and wellbeing indicators. Results found that all three resources were significant mediators for mental health and satisfaction with life); whereas only optimism was a significant mediator for physical health. Results are outlined in detail below. See Table 3 for the estimates of direct and indirect effects of the multiple mediation analysis.

**Table 3:** Direct and indirect effects of the mediation analyses.

Direct effects	*b*	*SE*	*Z*	*p*	95% BCaCI/CI
ACE-IQ → PCS	−2.04	0.33	−6.08	**	[−2.679, −1.367]
ACE-IQ → MCS	−1.85	0.31	−5.93	**	[−2.452, −1.230]
ACE-IQ → SWLS	−0.63	0.23	−2.74	.006*	[−1.062, −0.157]
Indirect effects					
Physical health					
ACE-IQ → Self-efficacy → PCS	−0.07	0.15	−0.45	.648	[−0.392, 0.211]
ACE-IQ → Optimism → PCS	−0.43	0.19	−2.25	.024*	[−0.882, −0.114]
ACE-IQ → Social support → PCS	−0.01	0.11	0.10	.915	[−0.210, 0.233]
Mental health					
ACE-IQ → Self-efficacy → MCS	−0.55	0.16	−3.35	**	[−0.946, −0.285]
ACE-IQ → Optimism → MCS	−0.85	0.21	−4.10	**	[−1.302, −0.501]
ACE-IQ → Social support → MCS	−0.35	0.11	−3.31	**	[−0.611, −0.180]
Satisfaction with life					
ACE-IQ → Self-efficacy → SWLS	−0.25	0.10	−2.50	.012*	[−0.491, −0.083]
ACE-IQ → Optimism → SWLS	−0.57	0.14	−4.05	**	[−0.892, −0.333]
ACE-IQ → Social support → SWLS	−0.31	0.08	−3.59	**	[−0.512, −0.169]

ACE-IQ, adverse childhood experiences—international questionnaire (early-life adversity indicator); *b*, estimate; MCS, mental component summary (mental health indicator); PCS, physical component summary (physical health indicator); *SE*, robust standard error; SWLS, satisfaction with life scale; *Z*, *z*-value; 95% BCaCI, bias-corrected and accelerated bootstrapped confidence interval for indirect effects; 95% CI, confidence interval for direct effects; *p*, *p*-value.

* *p* < .05.

** *p* < .001.

#### 3.3.1. Physical health

Regarding ELA and physical health, a significant indirect effect was found for optimism (*b* = −0.43, *SE *= 0.19, *p *= .024). No significant indirect effects were found for self-efficacy (*b* = −0.07, *SE *= 0.15, *p *= .648) or social support (*b* = −0.01, *SE *= 0.11, *p *= .915). As the direct effect between ELA and physical health remained significant (*b* = −2.04, *SE *= 0.33, *p *< .001) the results indicate that optimism partially mediated the association between ELA and physical health.

#### 3.3.2. Mental health

Regarding ELA and mental health, significant indirect effects were found for self-efficacy (*b* = −0.55, *SE *= 0.16, *p *< .001), optimism (*b* = −0.85, *SE *= 0.21, *p *< .001), as well as social support (*b* = −0.35, *SE *= 0.11, *p *< .001). As the direct effect between ELA and mental health remained significant (*b* = −1.85, *SE *= 0.31, *p *< .001), the results indicate that self-efficacy, optimism, and social support partially mediated the association between ELA and mental health.

#### 3.3.3. Satisfaction with life

Regarding ELA and satisfaction with life, significant indirect effects were found for self-efficacy (*b* = −0.25, *SE *= 0.10, *p *= .012), optimism (*b* = −0.57, *SE *= 0.14, *p *< .001), as well as social support (*b* = −0.31, *SE *= 0.08, *p *< .001). As the direct effect between ELA and satisfaction with life remained significant (*b* = −0.63, *SE *= 0.23, *p *= .006), results indicate that self-efficacy, optimism, and social support partially mediated the association between ELA and satisfaction with life.

### 3.4. Moderated mediation analysis

**Figure 1:**
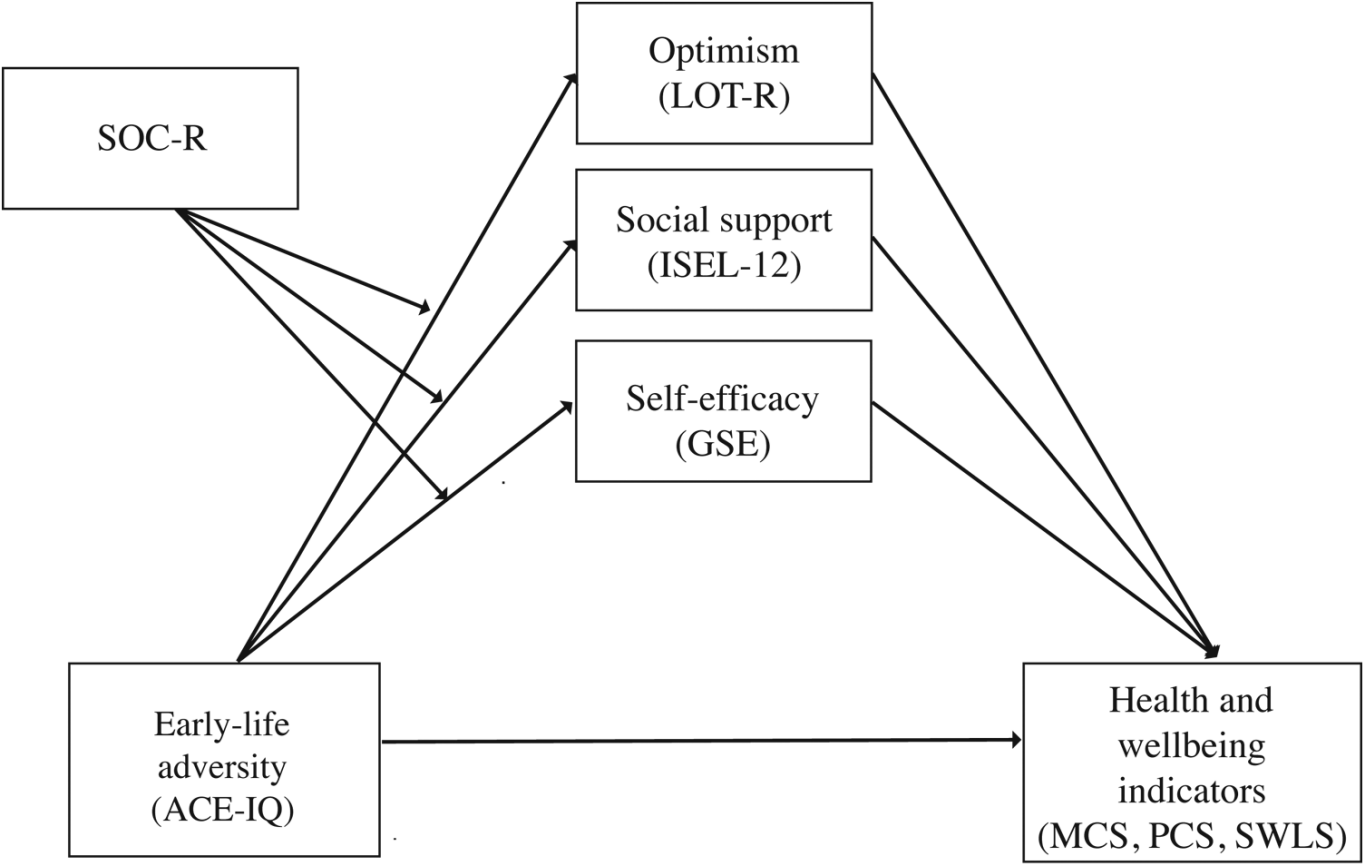
The proposed moderated mediation model, with SOC-R moderating the indirect effects of self-efficacy, optimism, and social support on early-life adversity and health and wellbeing. ACE-IQ, Adverse Childhood Experiences—International Questionnaire; GSE, general self-efficacy scale; ISEL-12, 12-item short form interpersonal support evaluation list; LOT-R, life orientation test—revised; MCS, mental component summary; PCS, physical component summary; SOC-R, sense of coherence—revised; SWLS, satisfaction with life scale.

In the next step, moderated mediation analyses were conducted to investigate whether SOC-R moderated the significant indirect effects from the previous mediation analyses. Results found significant indirect effects for all health and wellbeing indicators (i.e., physical and mental health, satisfaction with life) when SOC-R was included as a moderator, with the indirect effects larger at high rather than low levels of SOC-R. Results are outlined in detail below. Figure 1 illustrates the proposed moderated mediation model.

**Figure 2:**
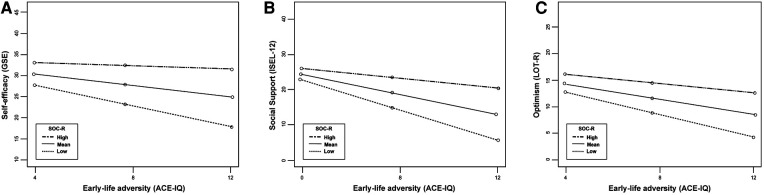
Significant moderation by SOC-R in the relationships between early-life adversity and the resources (**A**: self-efficacy, **B**: social support, **C**: optimism). ACE-IQ, Adverse Childhood Experiences—International Questionnaire; GSE, general self-efficacy scale; ISEL-12, 12-item short form interpersonal support evaluation list; LOT-R, life orientation test—revised; SOC-R, sense of coherence—revised; high and low SOC-R refer to one standard deviation above and below the mean.

First, it was tested whether SOC-R moderated the relationships between ELA and the three mediators (i.e., self-efficacy, optimism, social support). SOC-R significantly moderated the associations between ELA and self-efficacy (*b *= .06, *t* = 3.65*, p* = .001), between ELA and optimism (*b *= .04, *t* = 2.60, *p* = .009), and between ELA and social support (*b* = .08, *t* = 3.75, *p* < .001), such that the negative association between ELA and the resources became weaker at high levels of SOC-R. This allowed for the testing of the indirect effects of the relationships between ELA and the health and wellbeing indicators for conditional dependence on SOC-R. See Table 4 for the conditional indirect effects across all health and wellbeing indicators. See Figure 2 for the significant moderations by SOC-R of the relationship between ELA and the three mediators (i.e., self-efficacy, optimism, social support).

**Table 4:** Conditional indirect effects for the moderator SOC-R.

Predictor: ELA	Conditional indirect effects					
	High SOC-R			Low SOC-R		
	*b*	95% BCaCI	*p*	*b*	95% BCaCI	*p*
Mental health						
Self-efficacy	1.73	[0.534, 2.980]	.010*	1.04	[0.217, 1.900]	.018*
Optimism	1.44	[0.098, 2.970]	.038*	0.73	[−0.250, 1.715]	.160
Social support	0.74	[0.261, 1.233]	**	0.70	[−0.423, 1.766]	.196
Physical health						
Optimism	0.33	[0.005, 0.693]	.052	0.17	[−1.380, 1.102]	.800
Satisfaction with life						
Self-efficacy	0.51	[0.242, 0.811]	**	0.18	[−0.607, 0.920]	.644
Optimism	0.50	[0.116, 0.852]	.004*	0.16	[−0.569, 0.851]	.632
Social support	0.58	[0.243, 0.921]	**	0.10	[−0.629, 0.792]	.868

*b*, unstandardized indirect effects based on 5,000 bootstrap samples, ELA, early-life adversity assessed by the adverse childhood experiences—international questionnaire; SOC-R, sense of coherence—revised; high and low SOC-R refer to one standard deviation above and below the mean; 95% BCaCI, bias-corrected and accelerated confidence interval; *p*, *p*-value.

* *p* < .05.

** *p* < .001.

#### 3.4.1. Physical health

Regarding the partial mediation of ELA and physical health, the indirect effect of optimism was only marginally significant at high levels of SOC-R (*b* = 0.33, 95% BCaCI [0.005, 0.693], *p* = .052), and not significant at low levels of SOC-R (*b* = −0.17, 95% BCaCI [−1.380, 1.102], *p* = .800). The indirect effect of optimism showed an average increase of 94% at high (compared to low) levels of SOC-R. This could cautiously indicate that the mediating effect of optimism on the relationship between ELA and physical health was stronger when participants reported high levels of SOC-R. However, caution should be emphasized given that the *p*-value is very close to the standard significance threshold of .05.

#### 3.4.2. Mental health

Regarding the partial mediation of ELA and mental health, the indirect effects of self-efficacy were significant at high and low levels of SOC-R, with the size of the indirect effects being larger for high levels of SOC-R (*b *= 1.73, 95% BCaCI [0.534, 2.980], *p* = .010), rather than low levels of SOC-R (*b *= 1.04, 95% BCaCI [0.217, 1.900], *p* = .018). The indirect effect of self-efficacy showed an average increase of 40% at high (compared to low) levels of SOC-R. For optimism, the indirect effect was significant at high levels of SOC-R (*b *= 1.44*,* 95% BCaCI [0.098, 2.970], *p* = .038), but not at low levels of SOC-R (*b *= 0.73, 95% BCaCI [−0.250, 1.715], *p* = .160). The indirect effect of optimism showed an average increase of 49% at high (compared to low) levels of SOC-R. Similarly for social support, the indirect effect was significant at high levels of SOC-R (*b *= 0.74, 95% BCaCI [0.261, 1.233], *p* < .001), but not at low levels of SOC-R (*b *= 0.70, 95% BCaCI [−0.423, 1.766], *p* = .196). The indirect effect of social support showed an average increase of 5% at high (compared to low) levels of SOC-R. The indirect effects of self-efficacy and optimism were on average 1.6 times larger, and social support was 1.05 times larger, at high levels of SOC-R than at low levels of SOC-R. This indicates that the mediating effects of these resources on the relationship between ELA and mental health were stronger when participants reported high levels of SOC-R.

#### 3.4.3. Satisfaction with life

Regarding the partial mediation of ELA and satisfaction with life, the indirect effects of self-efficacy were significant at high levels of SOC-R (*b *= 0.51, 95% BCaCI [0.242, 0.811], *p* < .001), but not at low levels of SOC-R (*b *= 0.18, 95% BCaCI [−0.607, 0.920], *p* = .644). The indirect effect of self-efficacy showed an average increase of 64% at high (compared to low) levels of SOC-R. This pattern was also found for optimism (high SOC-R: *b *= 0.50, 95% BCaCI [0.116, 0.852], *p* = .004; low SOC-R: *b *= 0.16, 95% BCaCI [−0.569, 0.851], *p* = .632); and social support (high SOC-R: *b *= 0.58, 95% BCaCI [0.243, 0.921], *p* < .001; low SOC-R: *b *= 0.10, 95% BCaCI [−0.629, 0.792], *p* = .868). The indirect effect of optimism showed an average increase of 68% and indirect effect of social support showed an average increase of 82% at high (compared to low) levels of SOC-R. Across the three mediators, the indirect effects were on average 3.9 times larger at high levels of SOC-R than at low levels of SOC-R. This indicates that the mediating effects of self-efficacy, optimism, and social support on the relationship between ELA and satisfaction with life were stronger when participants reported high levels of SOC-R.

## 4. Discussion

This study examined the role of SOC-R and selected individual and external resilience-related resources in the relationship between early-life adversity and later-life health and wellbeing. A conditional process analysis was conducted, with a multiple mediation analysis performed in a first step, to test the indirect effects of self-efficacy, optimism, and social support as parallel mediators of the relationship between early-life adversity and indicators of health and wellbeing. In a second step, a moderated mediation tested for conditional dependence on SOC-R (i.e., the moderator). The main findings suggest that a strong SOC-R may have a beneficial influence on health and wellbeing by mitigating the detrimental effect of early-life adversity on the resources self-efficacy, optimism, and social support. Specifically, the analysis revealed significant partial mediations for all three resilience-related resources, with significant indirect effects when SOC-R was included as a moderator. The indirect effects were larger at high rather than low levels of SOC-R, indicating that the mediating effects of the resources on health and wellbeing were greater for participants with a strong SOC-R.

With regard to the mediation in the first step, self-efficacy, optimism, and social support were all found to partially mediate the relationship between early-life adversity and mental health, as well as satisfaction with life. While the partial mediation hints at the potential influence of additional resources, the findings are consistent with existing literature on the protective effects of psychosocial resources for those with a history of early-life adversity [e.g., (21, 57, 69)]. However, in relation to physical health, only optimism was shown to be a significant mediator in the present study. While research on early-life adversity and resilience-related resources often focuses on mental health (70), studies have also identified a positive influence of resources on physical health. For instance, research by Sachs-Ericsson and colleagues (71) found that self-efficacy significantly mediated the relationship between childhood abuse and the number of current physical health problems in a sample of older adults. One explanation for this discrepancy may be differences in the operationalization of physical health. The present study employed a subjective self-assessment of physical health rather than an objective or more impartial indicator, such as the number of medical conditions, cardiovascular risk factors (e.g., smoking status, waist-to-hip ratio), or hospitalizations [e.g., (7, 71, 72)]. Another possible explanation for the significant mediation by optimism, may be that optimism is a more vital resource than self-efficacy or social support in relation to physical health. Studies have consistently shown that individuals with higher levels of optimism are more likely to engage in health-promoting behaviors, such as having a healthier diet, being more physically active, and being a non-smoker (73, 74). However, with limited research on resilience-related resources and physical health after early-life adversity, further investigation is required to elucidate the underlying mechanisms for physical health.

Regarding the moderated mediation in the second step, the inclusion of SOC-R into the model resulted in a significant moderation of the relationship between early-life adversity and all three resources. In support of the hypothesis, the mediating effects of the resources were greater for participants with a stronger SOC-R, compared to those with a weaker SOC-R. This suggests that a strong SOC-R may have a beneficial influence on (mental) health by mitigating the detrimental effect of early-life adversity on the resources of self-efficacy, optimism, and social support. These results are also in line with research on the original SOC construct, which showed higher SOC to be linked to higher levels of mental health-related resources [e.g., (30, 75, 76)].

Within the moderated mediation, the largest mediating effects were observed for satisfaction with life, which were on average 3.9 times larger for high SOC-R across the three resources (self-efficacy, optimism, social support). The inclusion of these resources expands on the initial studies on SOC-R, which established a positive association with life satisfaction in the context of (chronic and acute) stress and adversity (35, 36). It is also consistent with earlier research on SOC in older adulthood, such as the study by Wiesmann and Hannich (77), which found that SOC pooled the influence of physical health, everyday competence, social support, and self-esteem on general life satisfaction and satisfaction with health in older age. The findings could suggest that a strong SOC-R may be beneficial in relation to successful ageing processes. For instance, the selective optimization with compensation theory proposes that older adults can foster successful ageing by identifying or reprioritizing goals on which to focus resources and adapting to limitations (78). The current results may indicate that SOC-R could play a role in this process, as those with a strong SOC-R could better identify and utilize their resources to maintain satisfaction with life in older age (28, 35). Future longitudinal studies could assess whether differences in the level of SOC-R are linked to the application of the life management strategies selection, optimization, and compensation [e.g., (79, 80)]; and ultimately, to variations in resource utilization and health outcomes over time.

Regarding the mediating effects for mental health, the indirect effects at high levels of SOC-R were slightly larger for optimism and self-efficacy, followed by social support. This may indicate that for mental health, having a strong SOC-R is particularly relevant for the protective influence of individual (i.e., optimism, self-efficacy) rather than external (i.e., social support) resources. Social support may also have a reduced influence in this particular sample due to the history of early-life adversity, as studies have shown links to lower levels of social support and interpersonal difficulties in adulthood [e.g., (81)]. The current study also assessed overall perceived social support, whereas a more nuanced indicator of social support (e.g., structural, tangible, emotional) may reveal more specific findings regarding the effect on mental health. However, social support should not be overlooked, given the significant indirect effects on both mental health and satisfaction with life in the present study. SOC-R and perceived social support may represent important targets for intervention in this older adult cohort, given the potential for alterations in social functioning [e.g., trust, attachment, or interpersonal issues (82)] in those who experienced early-life adversity; combined with the generally reduced social network in later life [e.g., due to death, reduced mobility, or entry into care home (83)].

Regarding physical health, while the mediating effect of optimism was larger at high levels of SOC-R, this was only marginally significant. These findings on mental and physical health are consistent with the existing literature on SOC-R, which has previously demonstrated associations with mental health, but found limited evidence for a connection with physical health [e.g., (36)]. Similarly, research with the original SOC construct has most often identified strong associations with indicators of positive mental health, rather than physical health (84). For example, a study by Galletta and colleagues (85) assessed the relationship between SOC and health-related quality of life in adults with chronic illnesses. Results found that SOC was directly correlated with mental health, but not the physical health component of quality of life. Rather, SOC showed an indirect effect on physical health through mental health, with the authors concluding that SOC is a psychological process most relevant for mental health-related quality of life (85). However, given the limited research on the revised SOC-R and physical health, further studies should examine this relationship in more detail, with the inclusion of both objective and subjective physical health indicators.

The results of this study highlight several implications and recommendations for research and practice within the field of (mental) health promotion. As the first step in exploring the relationship between SOC-R and resilience-related resources after early-life adversity, the current analysis included self-efficacy, optimism, and social support as well-established psychosocial resilience resources [e.g., (21, 32)]. Given the partial mediating effects of these resources, future studies should expand this model to include additional internal and external psychosocial resources, such as self-esteem, self-compassion, coping beliefs, and attachment (25, 31, 86); as well as neurobiological factors, such as cortisol reactivity, inflammatory (dys-)regulation, and hypothalamic-pituitary-adrenal (HPA) axis function (26, 75). Going beyond interpersonal resources, consideration of the broader cultural and socio-ecological influences on SOC-R could include factors such as cultural value orientations, community cohesion, and utilization of health services (16, 21, 87). Such cultural and societal factors can influence how an individual processes adversity and activates coping resources (17). For example, stigma or silence surrounding child abuse, as well as negative stereotypes and expectations about masculinity, may reduce the likelihood that individuals, particularly men, seek help or access mental health services (75, 88). Replication of this study in other cultures and contexts, with a focus on potential gender differences, may reveal unique relationships between resources that could inform (gender- or culture-specific) targets for intervention [e.g., (89)]. Nevertheless, the current findings identify SOC-R and the psychosocial resources self-efficacy, optimism, and social support as potential intervention targets for older adults affected by early-life adversity, particularly in relation to mental health and satisfaction with life.

Regarding practice implications, therapeutic intervention approaches that aim to strengthen SOC-R may improve individuals' awareness and utilization of their internal and external resources. For example, a group-based approach to narrative therapy aiming to increase sense of coherence may help to foster a positive self-identity, enhance social support, and promote confidence in coping through resource use by providing a supportive environment for self-reflection and shared coping narratives (90). Applying a salutogenic orientation to health promotion, clinicians could utilize salutogenic dialogue for the improvement of health literacy, i.e., increasing patients' awareness of their strengths, resources, and capabilities with regard to managing their own health conditions [e.g., (91)]. This could enhance patient wellbeing and help facilitate a paradigm shift towards preventive intervention and a salutogenic model of healthcare.

Furthermore, consideration of the limitations of the present study can also provide direction for future research. First, it should be emphasized that this study applied a cross-sectional, retrospective design, which precludes the establishment of causal relations. To provide stronger evidence for causality, future studies using longitudinal, prospective designs should investigate if similar moderating and mediating effects can be observed, exploring the causal directionality of SOC-R, resources, and health and wellbeing. Second, although attempts were made to ensure the sample was proportioned to the general population (40), these findings represent the target sample of Irish (older) adults with experiences of early-life adversity and may therefore not be generalizable. Future research could examine resilience-related resources and (mental) health in younger samples or in larger nationally representative surveys. Third, the scoring of the PCS and MCS as distinct physical and mental dimensions of health status may mask findings on the subscale level, as well as potential links between physical and mental health. Future studies could benefit from the inclusion of more nuanced or correlated indicators of physical and mental health. Fourth, the subjective self-assessment of physical health used in the present study may limit the interpretation of the physical health findings. Future research could include more objective or impartial indicators of physical health to provide a more comprehensive understanding of the relationship between resilience-related resources and physical health. Last, the measure of early-life adversity employed in the present study encompassed several adversity types, including abuse, neglect, and witnessed domestic violence (1). While this provides a comprehensive assessment of early-life adversity, the specific adversity types may have vastly different impacts on (mental) health and wellbeing [e.g., (92)]. In examining the influence of SOC-R and resilience-related resources, future studies could examine the impact of distinct adversity types (e.g., sexual, physical, and emotional abuse; physical and emotional neglect; witnessed violence). In addition, as the current study focused on individual and interpersonal resources, future studies could build on these findings to explore the relationship between SOC-R and broader cultural or socio-ecological resources, such as cultural norms and values, shared religious and spiritual beliefs, or community structures and resources (16, 17). Nevertheless, despite these limitations, the present study represents a useful addition to the literature on the role of SOC-R and resilience-related resources in the relationship between early-life adversity and later-life (mental) health. It also provides preliminary empirical support for the theoretical assumptions of SOC-R, namely, that a strong SOC-R can facilitate the utilization of available and appropriate resources to overcome adversity (28).

### 4.1. Conclusion

Key findings from the present study suggest that a strong SOC-R may have a beneficial influence on health and wellbeing by mitigating the detrimental effect of early-life adversity on the resources self-efficacy, optimism, and social support. The effects of these resilience-related resources were greater for individuals with a strong (rather than weak) SOC-R. Defined as the ability to perceive, integrate, and balance life experiences in order to facilitate health maintenance; SOC-R was particularly influential for mental health and life satisfaction in this older adult sample with a history of early-life adversity. Regarding the implications, the findings can also highlight potential impacts on the field of mental health promotion. For instance, having a strong SOC-R could be important for healthy or successful ageing, with an avenue for future research focusing on the potential role of SOC-R in the selection and adaptation of goals and resources into older age. Furthermore, given its moderating role between early-life adversity and psychosocial resources, SOC-R may represent a promising target for inclusion in psychotherapeutic interventions. For example, enhancing SOC-R may help to improve awareness and utilization of resources as a pathway to mental health and wellbeing and promote resilience in survivors of childhood trauma or adversity. In sum, by providing initial evidence for a beneficial role of SOC-R and the resilience-related resources self-efficacy, optimism, and social support, this study contributes to the literature and adds to the understanding of the mechanisms underpinning the pathways to (mental) health and wellbeing following early-life adversity.

## Data Availability

The datasets presented in this study can be found in online repositories. The names of the repository/repositories and accession number(s) can be found below: The data that support the findings of this study are openly available in the Open Science Framework (OSF) at http://doi.org/10.17605/OSF.IO/K4H2U, identifier: DOI 10.17605/OSF.IO/K4H2U.
